# Harvesting wildlife affected by climate change: a modelling and management approach for polar bears

**DOI:** 10.1111/1365-2664.12864

**Published:** 2017-03-08

**Authors:** Eric V. Regehr, Ryan R. Wilson, Karyn D. Rode, Michael C. Runge, Harry L. Stern

**Affiliations:** ^1^ U.S. Fish and Wildlife Service Anchorage AK USA; ^2^ U.S. Geological Survey Anchorage AK USA; ^3^ U.S. Geological Survey Laurel MD USA; ^4^ University of Washington Seattle WA USA; ^5^ Present address: University of Washington Seattle WA USA

**Keywords:** conservation, density dependence, habitat loss, harvest, hunting, polar bear *Ursus maritimus*, risk, state‐dependent management, sustainable, threatened

## Abstract

1. The conservation of many wildlife species requires understanding the demographic effects of climate change, including interactions between climate change and harvest, which can provide cultural, nutritional or economic value to humans.
2. We present a demographic model that is based on the polar bear *Ursus maritimus* life cycle and includes density‐dependent relationships linking vital rates to environmental carrying capacity (*K*). Using this model, we develop a state‐dependent management framework to calculate a harvest level that (i) maintains a population above its maximum net productivity level (MNPL; the population size that produces the greatest net increment in abundance) relative to a changing *K*, and (ii) has a limited negative effect on population persistence.
3. Our density‐dependent relationships suggest that MNPL for polar bears occurs at approximately 0·69 (95% CI = 0·63–0·74) of *K*. Population growth rate at MNPL was approximately 0·82 (95% CI = 0·79–0·84) of the maximum intrinsic growth rate, suggesting relatively strong compensation for human‐caused mortality.
4. Our findings indicate that it is possible to minimize the demographic risks of harvest under climate change, including the risk that harvest will accelerate population declines driven by loss of the polar bear's sea‐ice habitat. This requires that (i) the harvest rate – which could be 0 in some situations – accounts for a population's intrinsic growth rate, (ii) the harvest rate accounts for the quality of population data (e.g. lower harvest when uncertainty is large), and (iii) the harvest level is obtained by multiplying the harvest rate by an updated estimate of population size. Environmental variability, the sex and age of removed animals and risk tolerance can also affect the harvest rate.
5. *Synthesis and applications*. We present a coupled modelling and management approach for wildlife that accounts for climate change and can be used to balance trade‐offs among multiple conservation goals. In our example application to polar bears experiencing sea‐ice loss, the goals are to maintain population viability while providing continued opportunities for subsistence harvest. Our approach may be relevant to other species for which near‐term management is focused on human factors that directly influence population dynamics within the broader context of climate‐induced habitat degradation.

## Abstract 2

We present a coupled modelling and management approach for wildlife that accounts for climate change and can be used to balance trade‐offs among multiple conservation goals. In our example application to polar bears experiencing sea‐ice loss, the goals are to maintain population viability while providing continued opportunities for subsistence harvest. Our approach may be relevant to other species for which near‐term management is focused on human factors that directly influence population dynamics within the broader context of climate‐induced habitat degradation.

## Introduction

Climate change will be an important driver of biodiversity loss into the foreseeable future. Habitat degradation, phenological shifts and ecosystem change are expected to result in an increasing number of species of conservation concern (Walther *et al*. 2002). Improved methods are needed to address the interactive effects of climate change and other, direct human impacts on wildlife populations. Such methods can be used to evaluate the effectiveness of management and recovery actions, and to balance the costs and benefits inherent to conservation planning.

The Arctic has warmed at approximately twice the global rate causing declines in the extent, temporal availability and thickness of sea ice (IPCC 2014). Arctic marine mammals depend on sea ice for many aspects of their life history and some are particularly vulnerable due to specialized feeding or habitat requirements (Laidre *et al*. 2008). In 2008, the polar bear *Ursus maritimus* Phipps 1774 was listed as ‘threatened’ under the U.S. Endangered Species Act based on forecasted population declines due to observed and forecasted sea‐ice loss (USFWS 2008). Globally, polar bears are divided into 19 subpopulations that currently exhibit variable demographic status (Regehr *et al*. 2016). There is empirical evidence that two subpopulations have experienced sea‐ice related declines (Bromaghin *et al*. 2015; Lunn *et al*. 2016). Several subpopulations show signs of stress (Obbard *et al*. 2016) or have been reported as stable or productive (Stirling *et al*. 2011; Peacock *et al*. 2013; Rode *et al*. 2014; Stapleton, Peacock & Garshelis 2016), and others have unknown status due to deficient data (Obbard *et al*. 2010). Despite this variability, projected sea‐ice loss in the 21st century (Stroeve *et al*. 2012) is expected to negatively affect polar bears throughout much of their range, because the species depends fundamentally on sea ice for access to its primary prey (Atwood *et al*. 2016). Management and conservation planning will therefore require methods to consider both current population status as well as the anticipated effects of habitat loss. Planning must also consider reliance on imperfect population data due to the difficulties of studying animals that occur at low densities in remote environments (Laidre *et al*. 2015).

Wildlife managers will face new challenges in the 21st century because the agencies responsible for conservation planning may not have the ability to influence greenhouse gas emissions (e.g. USFWS 2013), the primary cause of anthropogenic climate change. Consequently, near‐term management actions will likely focus on secondary factors or threats with the intent of protecting populations until global action leads to a stabilized climate system (Seney *et al*. 2013), or until conservation priorities change. For polar bears, subsistence harvest is important because it has cultural, nutritional and economic value to Native people in the Arctic (e.g. Voorhees *et al*. 2014), and because it is a direct and controllable source of mortality. Fifteen polar bear subpopulations currently support a legal subsistence harvest (Laidre *et al*. 2015) through which approximately 735 animals are removed each year (Shadbolt, York & Cooper 2012) from a global population of approximately 26 000 (Wiig *et al*. 2015). This level of use is not considered a threat to polar bears at the species level (USFWS 2008; Atwood *et al*. 2016). However, there have been concerns about harvest for individual subpopulations (Obbard *et al*. 2010), and future interactions between climate change and human‐caused removals (i.e. the combination of subsistence harvest, defence kills and other direct sources of mortality) are less clear. For example, habitat loss could increase vulnerability to overutilization if populations become smaller or less resilient and removal levels are not adjusted accordingly. Habitat loss also could lead to increased human–bear conflicts if longer ice‐free seasons result in more nutritionally stressed polar bears on land (Towns *et al*. 2009). The need for a robust, science‐based framework to evaluate the combined effects of climate change and human‐caused removals is emphasized by social and political attention to the harvest of iconic species (e.g. Di Minin, Leader‐Williams & Bradshaw 2016).

In this paper, we present a demographic and management model that is based on the polar bear life cycle (Hunter *et al*. 2010; Regehr *et al*. 2010, 2015). The model includes density‐dependent relationships linking vital rates to a changing environmental carrying capacity (*K*) on the basis of polar bear biology, energetic requirements and population dynamics theory. Including density dependence is necessary to evaluate how populations will respond to concurrent changes in human‐caused mortality and *K* (Guthery & Shaw 2013), and we represent trends and variability in *K* using a proxy metric derived from remote sensing data for sea‐ice habitat (Stern & Laidre 2016). The model also includes simulated population assessments, so that management actions can be evaluated in light of imperfect population data and time lags. Next, we develop a state‐dependent (i.e. dependent on current conditions; Lyons *et al*. 2008) management framework, based on harvest theory and the potential biological removal method (Wade 1998; Runge *et al*. 2009), which accommodates the potential for habitat loss to affect populations through both density‐dependent and density‐independent mechanisms. Finally, we demonstrate how to use the demographic and management model to calculate inputs to the state‐dependent management framework, resulting in a harvest that meets user‐specified management objectives with a clear statement of risk tolerance. We also present a sample application of the state‐dependent management framework to simulated polar bear populations under a wide range of conditions including rapidly declining *K*.

## Materials and methods

### Demographic and management model

**Figure 1:**
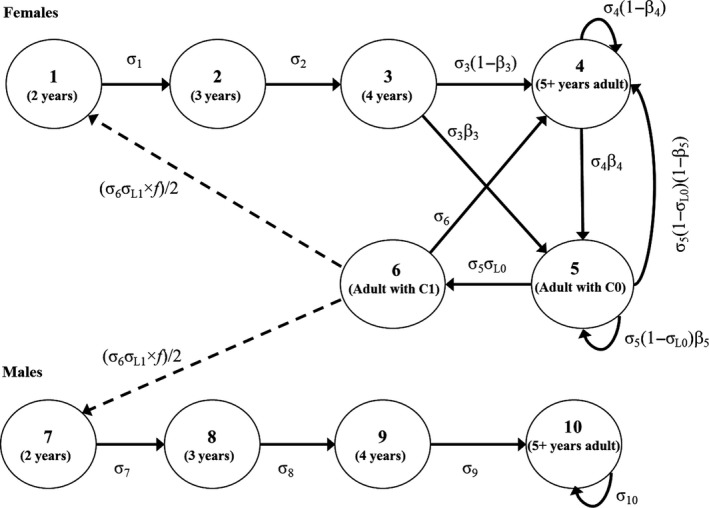
The polar bear life cycle graph underlying the matrix‐based projection model. Stages 1–6 are females and stages 7–10 are males; σ_*i*_ is the annual probability of survival of an individual in stage *i*, σ_L0_ and σ_L1_ are the probabilities of at least one member of a cub‐of‐the‐year (C0) or yearling (C1) litter surviving, *f* is the expected size of C1 litters that survive to 2 years, and β_*i*_ is the probability, conditional on survival, of an individual in stage *i* breeding, thereby producing a C0 litter with at least one member surviving. Solid lines are stage transitions and dashed lines are reproductive contributions.

We constructed a matrix‐based projection model that includes six female stages representing age and reproductive status, and four male stages representing age (Hunter *et al*. 2010; Regehr *et al*. 2010). Transitions between stages are defined by vital rates relative to a post‐breeding census from the spring of year *t* to the spring of year *t *+* *1 (Fig. 1; modified from Hunter *et al*. 2010 to allow a transition from stage 3 to 5). We used published vital rates from case studies for polar bears to inform some aspects of model development and analysis (see Appendix S1, Supporting Information).

#### Density dependence

**Figure 2:**
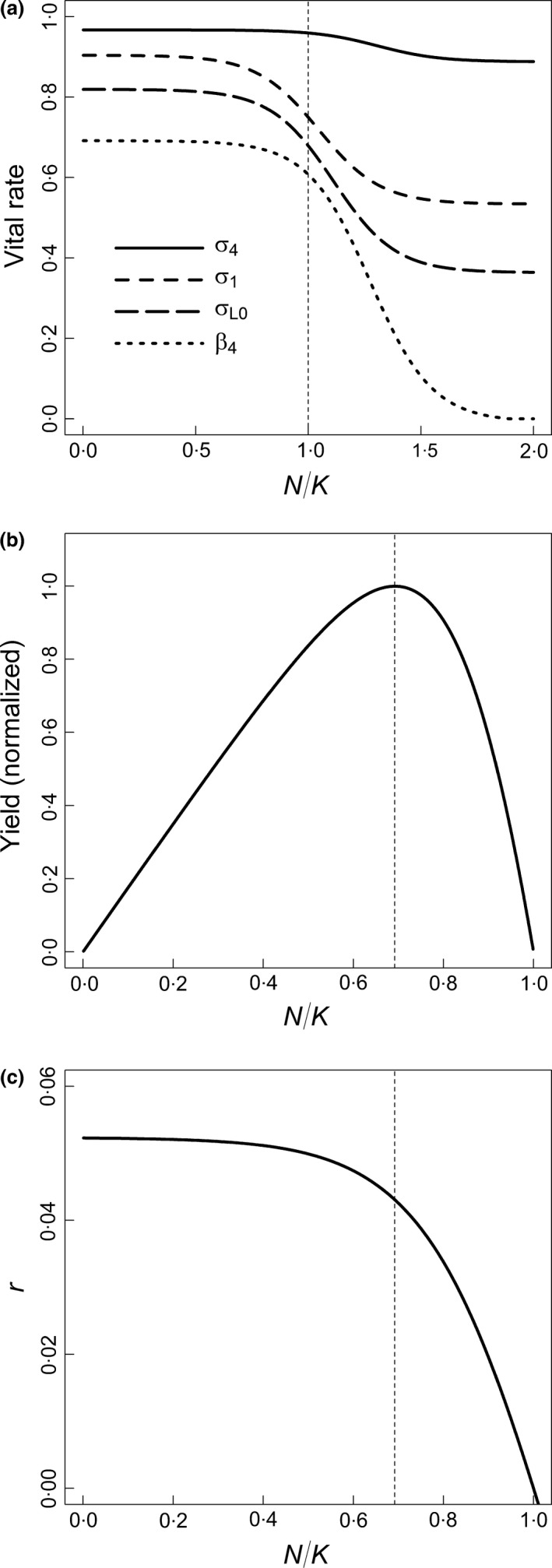
Sample graphs showing the model of density dependence for polar bears. Density is expressed as the ratio of population size (*N*) to carrying capacity (*K*) on the *x*‐axis. (a) The nonlinear density‐dependent curves of the vital rates. Vital rates are defined in Fig. 1. The vertical line corresponds to *N*/*K *=* *1 at carrying capacity. (b) The corresponding yield curve. The vertical line corresponds to *N*/*K *=* *0·69 at maximum net productivity level (MNPL). (c) The asymptotic per capita population growth rate (*r*). The vertical line corresponds to MNPL and intersects the curve at *r*_MNPL_. All graphs are for a population with medium resilience.

For long‐lived species, the relationships between vital rates and density are decreasing and likely convex (Williams 2013). Because empirical data on density dependence are lacking for polar bears (Derocher & Taylor 1994), we developed plausible density‐dependent curves using a logistic equation that was parameterized based on insights from population theory and evolutionary ecology (sample curves are shown in Fig. 2a; see Appendix S2). The relative positions of the inflection points in the curves reflect observations that density effects for large mammals typically appear first in subadult survival, then in breeding rates and juvenile survival, and finally in adult survival (Fowler 1987; Eberhardt 2002). For polar bears, adult female survival is the most important determinant of population growth (Eberhardt 1990). We based the magnitude of potential density‐related variation in adult female survival (the vital rates σ_4_, σ_5_ and σ_6_; Fig. 1) on the observed range of survival estimates from case studies. We then set the magnitude of density‐related variation in other vital rates to be inversely proportional to their elasticities, as calculated from the matrix model, relative to the mean elasticity of adult female survival. Elasticity reflects the proportional influence of a vital rate on population growth (Caswell 2001). Our approach therefore was a quantitative representation of the hypothesis of ‘demographic buffering’, under which the most important vital rates exhibit the least amount of density‐related variation (Pfister 1998).

Density dependence in polar bears is influenced by direct and indirect competition for nutritional resources (Derocher & Taylor 1994). Individual polar bears vary in body size and metabolic demands (Molnár *et al*. 2009), and thus potentially in their contributions to density effects. We incorporated the hypothesis that sex‐ and age‐specific energetic requirements influence population regulation (Stirling & Øritsland 1995) by calculating metabolic energetic equivalent values from field data for the body mass of polar bears in each stage of the life cycle graph (see Appendix S2). We accounted for dietary differences (e.g. due to the ability of males to access larger prey; Thiemann, Iverson & Stirling 2008) that could impact competitive interactions. We then represented population density as the sum of metabolic energetic equivalents for individuals in the population (rather than the simple sum of individuals) divided by *K* expressed as energetic equivalents.

The density‐dependent relationships underlie key population dynamics. For each set of curves, there is a unique density that produces a combination of vital rates resulting in a realized per capita population growth rate (*r*) equal to 0. By definition, this corresponds to a location on the *x*‐axis representing the equilibrium population size (*N*) at carrying capacity (*K*), at which the dimensionless ratio *N/K *=* *1·0 (Fig. 2a). Maximum net productivity level (MNPL) was defined as the population size corresponding to the ratio *N*/*K* at which the greatest net annual increment in abundance occurs, as shown on the corresponding yield curve (Fig. 2b). (In this paper, MNPL refers to the preceding scientific definition; it does not refer to the concept defined in US regulations under the Marine Mammal Protection Act, 50 CFR 216.3.). The per capita growth rates *r*_max_ and *r*_MNPL_ provide measures of a population's capacity for growth at small population sizes and at MNPL respectively (Fig. 2c). We use *r*_MNPL_ as a measure of a population's resilience, defined as its capacity to grow following a reduction to below *K*, in the absence of human‐caused removals.

#### Carrying capacity and environmental variation

Modelling wildlife populations under climate change requires considering the effects of variability and trends in the environment (Boyce *et al*. 2006). We derived a proxy metric for *K* using satellite data of sea‐ice extent for the Chukchi Sea (CS) and southern Beaufort Sea (SB) regions, which contain the two polar bear subpopulations that occur partially within US territory (see Appendix S3). First, we calculated the number of days per year (1979–2013) that sea ice covered ≥50% of the mean sea‐ice area in March, following the methods of Stern & Laidre (2016). Then, under several scenarios, we projected the proportional change in the number of ice‐covered days, relative to a baseline period, using annual time steps. The resulting dimensionless metric (κ) captured the variance and trend in the duration of the period that polar bears likely have greatest access to their prey. Carrying capacity at year *t*, calculated as *K*(*t*) = *K*(*t *=* *1) × κ(*t*), operated on vital rates through the density‐dependent relationships. We subjectively included additional density‐independent variation as 25% of total uncertainty in estimated vital rates from case studies, following the example of Taylor *et al*. (2002). Density‐independent variation was implemented using a correlation matrix calculated from annual estimates of vital rates for the SB subpopulation (see Appendix S3).

#### Harvest and simulated population assessments

We used the matrix model to perform population projections (see Simulations) in which harvest was implemented annually at a calculated level (measured in number of animals) that was updated at 10‐year management intervals. To account for selectivity in human‐caused removals and individual variation in reproductive value, the model included stage‐specific harvest vulnerabilities calculated from demographic and harvest data for the SB subpopulation (see Appendix S4). This suggested that subadults of both sexes (stages 1–3 and 7–9; Fig. 1) were twice as likely to be killed by humans, relative to their stage distribution, compared to adults (stages 4 and 10; Fig. 1). Harvest levels were calculated using estimates of vital rates and population size derived from simulated population assessments, which included different levels of data precision based on the amount of sampling uncertainty in case studies for polar bears (see Appendix S4).

### State‐dependent management framework

We present a state‐dependent management framework (eqns 1 and 2) for calculating a harvest that meets user‐specified management objectives. This framework extends the formula for allowable take in Runge *et al*. (2009), as follows:

(eqn 1) $$H^{\mathrm{female}}\left(t\right)=F_{O}\times {\tilde{r}}_{\mathrm{MNPL}}\left(t\right)\times 0.5\cdot .55\times \tilde{N}\left(t\right),\text{and}$$

(eqn 2) $$H^{\mathrm{male}}\left(t\right)=H^{\mathrm{female}}\left(t\right)\times SR$$

where *H* ^female^ is the number of females that can be removed annually; *F*_O_ is a factor that directly adjusts the harvest rate to reflect management objectives and the risk tolerance of managers with respect to harvest; ${\tilde{r}}_{\mathrm{MNPL}}$ is an estimate of the per capita population growth rate from population studies, referenced to population density at MNPL and selected from its sampling distribution to reflect risk tolerance; 0·5 is a factor to calculate female removals assuming an equal sex ratio in the population; $\tilde{N}$ is an estimate of population size (*N*) from population studies, selected from its sampling distribution to reflect risk tolerance; *H* ^male^ is the number of males that can be removed annually; and *SR* is a factor that specifies the male‐to‐female ratio in removals.

The notation for time (*t*) indicates that parameters are updated periodically as determined by the management interval. Equations 1 and 2 are written in terms of harvest level for convenience; the harvest rate for females is the right side of eqn 1 before multiplying by $\tilde{N}$.

Values of the management factor *F*_O_ can be evaluated from the results of population projections using the matrix model (see Simulations). Under ideal conditions, populations will stabilize at approximately MNPL when *F*_O_ = 1. Increasing *F*_O_ above 1 will result in an equilibrium population size of less than MNPL, until an upper limit on *F*_O_ is reached beyond which the removal rate exceeds the maximum per capita growth rate (*r*_max_) and drives *N* towards 0. This upper limit is *F*_O_ = 2 for the classic logistic model of population growth, but will vary based on life history (Williams 2013). The practical use of *F*_O_ is to direct a population towards a target size and to specify risk tolerance with respect to missing that target or achieving some other undesired outcome (e.g. extirpation). Such risks can result from stochasticity, uncertain information about population status and other imperfections in the modelling or management approach. Selection of ${\tilde{r}}_{\mathrm{MNPL}}$ and $\tilde{N}$ from within their sampling distributions will also affect the harvest level and thus the probability of meeting management objectives. The parameter *SR* can be used to implement sex‐specific removals, which is practical for polar bears because most harvests occur at a 2 : 1 male‐to‐female ratio (Taylor, McLoughlin & Messier 2008). Use of the term 0·5 in eqn 1, rather than a direct estimate of the female fraction of the population, provides an additional safeguard when harvest is male‐biased by calculating harvest rates as if only half of the population were female.

### Simulations

We projected hypothetical polar bear populations forward in time, subject to harvest, to identify threshold values for parameters in the state‐dependent management framework that meet our management objectives (see Appendix S5). Populations were projected over 50 annual time steps for all combinations of the following inputs:

1. Three levels of population resilience corresponding to *r*_MNPL_ = 0·015, 0·043 and 0·085; subsequently referred to as low, medium and high resilience respectively. These values were selected from the range of asymptotic *r* values estimated using vital rates (in the absence of harvest) from case studies for polar bears (mean *r *=* *0·05, 95% CI = 0·02–0·09; see Appendix S2).
2. Harvest implemented using 31 equal‐increment values of *F*_O_ from 0·5 to 1·25. We considered this range based on values that have been used for the conceptually related recovery factor in the PBR method (Wade 1998); other values could be used depending on management objectives.
3. Precision (levels 1 through 4) in the parameters estimated from simulated population assessments, corresponding to coefficients of variation (CV) for the estimated vital rate CV(σ_4_) = 0·003, 0·008, 0·018 and 0·089; and CV(*N*) = 0·04, 0·08, 0·15 and 0·25 (see Appendix S4). Additionally, we calculated harvest based on true values of the vital rates and *N*, updated annually, to illustrate the effects of harvest under perfect sampling and management (referred to as precision level ‘true’).
4. Three methods to select $\tilde{N}$ corresponding to the 5th, 15th and 50th percentiles of the sampling distribution for *N* from simulated population assessments. Using a lower percentile of *N* to calculate removal levels, as opposed to using the mean value, protects against overestimates when uncertainty is large.

For each combination of inputs we performed 1000 projections. Key outcomes were the probability of violating management objective (i), calculated as the proportion of projections for which *N* < MNPL at the final time step *t *=* *50 (i.e. *P*_<MNPL_); and the probability of persistence (i.e. *P*_persist_), calculated as the proportion of projections for which *N* never crossed below a quasi‐extinction threshold of 15% of starting population size. We defined *P*_persist_ relative to a proportional quasi‐extinction threshold due to the potential for negative small‐population dynamics such as Allee effects in the mating system (Molnár, Lewis & Derocher 2014; see Appendix S5).

To be fully specified in the face of uncertainty, management objectives need to include a risk tolerance. We inferred an upper limit on *P*_<MNPL_ as the value corresponding to a 10% decrease in *P*_persist_, relative to an identical projection that did not include harvest (see Appendix S5). This is strictly a placeholder degree of risk tolerance for demonstration. All projections started with a stable stage distribution at MNPL with respect to *K*(*t *=* *1) = 1000 animals and included interannual variability in the proxy for *K*, with the mean temporal trend in *K* set to 0 (see Appendix S3). The parameter ${\tilde{r}}_{\mathrm{MNPL}}$ was selected as the 50th percentile of the sampling distribution for *r*_MNPL_. We included an additional management rule constraining ${\tilde{r}}_{\mathrm{MNPL}}$ ≤0·10 to protect against excessive harvest rates when per capita growth rates were high and data precision was low. Harvest levels, implemented using *SR* = 2 and data‐based harvest vulnerability, remained constant over 10‐year management intervals.

We also present a sample application of the state‐dependent management framework, using a single set of inputs to eqns 1 and 2, to evaluate its performance under a wide range of conditions including declining *K*. The key outcome for this application was Δ*P*_extirpation_, the incremental change in the probability of extirpation (i.e. 1−*P*_persist_) for identical projections with and without harvest. Projections were performed for all combinations of the following inputs:

1. Four hundred sets of vital rates representing a plausible parameter space that encompasses estimates of vital rates from case studies for polar bears (Table S1).
2. Three rates of change in the proxy for *K*: (i) no temporal trend (i.e. stable carrying capacity), (ii) −7% per decade, approximately the mean trend in the proxy for *K* as estimated from sea‐ice data for the CS and SB regions; and (iii) −14% per decade.
3. Two approaches to harvest: (i) no harvest; and (ii) harvest using *F*_O_ = 0·75, *SR* = 2, and $\tilde{N}$ selected as the 15th percentile of the sampling distribution for *N*, with a data precision level of 3. These inputs to the state‐dependent management framework correspond broadly to the historic standard 4·5% harvest rate for polar bears (Taylor *et al*. 1987) when applied to the mean vital rates from case studies.

Simulations were performed using the R computing language (version R 3.1.0; The R Project for Statistical Computing; http://www.r-project.org; see Appendix S6).

## Results

The density‐dependent relationships resulted in a mean ratio of *N*/*K* at MNPL = 0·69 [95% confidence interval (CI) = 0·63–0·74] across the 400 sets of vital rates in the parameter space. MNPL was negatively correlated with survival and recruitment, such that populations with higher vital rates produced maximum sustainable yield at smaller equilibrium sizes relative to *K* (Fig. S1). The mean value of the ratio *r*_MNPL_/*r*_max_ was 0·82 (95% CI = 0·79–0·84).

**Figure 3:**
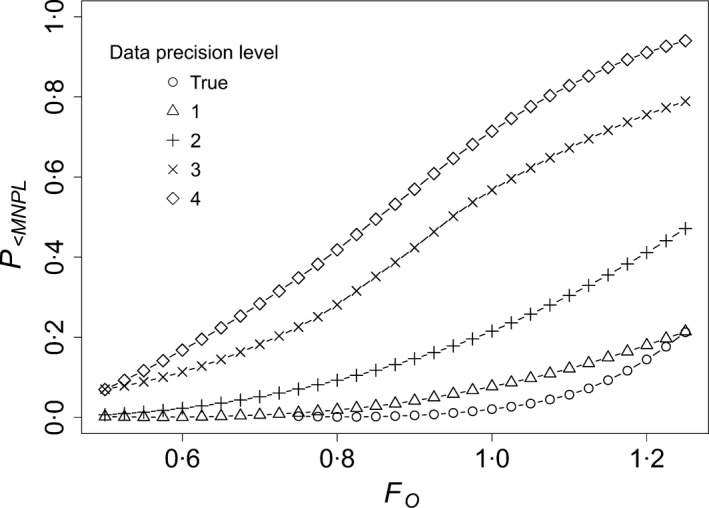
The probability that harvest will result in a population size less than the maximum net productivity level (*P*_*<*_ _MNPL_) as a function of the management factor (*F*_O_) in the state‐dependent management framework. Data precision levels refer to the amount of sampling uncertainty in estimates of vital rates and population size used to inform management (see Simulations). Results are shown for a population with medium resilience. Estimates of $\tilde{N}$ were selected as the lower 15th percentile of the sampling distribution for *N*.

The placeholder degree of risk tolerance, which specified that harvest should not decrease the probability of persistence by more than 10% over 50 years, required that the probability of a population falling below MNPL (i.e. *P*_<MNPL_) not exceed 0·22, 0·36 and 0·62 for populations with high, medium and low resilience respectively. Threshold values for *F*_O_, defined as the values necessary to remain below these upper limits on *P*_<MNPL_, were a function of population resilience, data precision and the approach used to select $\tilde{N}$ from its sampling distribution (Table 1). Human‐caused removals that are implemented using the proposed state‐dependent management framework, with a 10‐year management interval and values of *F*_O_ less than those in Table 1, would be expected to meet our management objectives based on the placeholder degree of risk tolerance. As expected, *P*_<NMPL_ was positively related to the management factor *F*_O_ (i.e. population declines are more likely when harvest is high) and negatively related to data precision (i.e. population declines are less likely when population data are better; Fig. 3).

**Table 1:** Threshold values of the management factor (*F*_O_) that meet management objectives, based on a placeholder degree of risk tolerance. Population resilience is defined in terms of the unharvested per capita population growth rate referenced to maximum net productivity level (*r*_MNPL_). Data precision levels are defined in the text. Population size (*N*) was selected from its sampling distribution using the lower 5th, 15th or 50th percentiles

Data precision level	*N* lower 5th percentile	*N* lower 15th percentile	*N* 50th percentile
(a) Low resilience (*r*_MNPL_ = 0·015)			
True	1·18	1·18	1·19
1	>1·25	>1·25	1·15
2	1·13	1·07	0·99
3	0·82	0·72	0·61
4	<0·50	<0·50	<0·50
(b) Medium resilience (*r*_MNPL_ = 0·043)			
True	>1·25	>1·25	>1·25
1	>1·25	>1·25	>1·25
2	1·21	1·15	1·07
3	0·96	0·86	0·72
4	0·95	0·76	0·56
(c) High resilience (*r*_MNPL_ = 0·085)			
True	>1·25	>1·25	>1·25
1	>1·25	>1·25	1·23
2	>1·25	1·22	1·12
3	>1·25	1·17	1·00
4	>1·25	1·11	0·82

Our sample application of the state‐dependent management framework with *F*_O_ = 0·75, *SR* = 2 and a data precision level of 3, resulted in a harvest that met management objectives over a wide range of biological conditions (Table 2). Increased risk of extirpation compared to no harvest was greatest for populations with *r*_MNPL_ in the range of −0·05–0. Such populations can only decline and any human‐caused mortality would be additive. The increased risk of extirpation associated with harvest was largely due to sampling error: the chance of a positively biased estimate of *r*_MNPL_ leading to an overestimate of the harvest rate that meets management objectives. Although the combination of *r*_MNPL_ ≤0 and stable *K* is probably not biologically realistic, it was included to illustrate the potential risks of human‐caused removals under severe density‐independent limitation. For populations with *r*_MNPL_ in the range 0–0·025, values of Δ*P*_extirpation_ ranged from 4% to 8%, depending on the trend in *K*, consistent with the placeholder degree of risk tolerance. Compared to populations with stable carrying capacity, values of Δ*P*_extirpation_ were similar for populations that experienced the mean estimated change of −7% per decade in the proxy for *K* (Table 2). This is because harvest was specified using a management objective relative to current *K*, which resulted in equilibrium sizes for harvested populations that declined in parallel to *K* but not at an accelerated rate. To illustrate, across all scenarios in Table 2 with declining *K*, the median rate of linear decline in *N* was indistinguishable for scenarios with and without harvest (mean difference in rate of decline −0·01 bears per year, SD = 1·3 bears per year). Values of Δ*P*_extirpation_ were moderately higher for populations that experienced a −14% change per decade in *K* (Table 2). This was largely because *K* declined so rapidly that small‐population dynamics became important towards the end of the 50‐year projection. That is, populations with smaller equilibrium sizes due to harvest were likely to cross the quasi‐extinction threshold sooner, due to stochasticity, compared to un‐harvested populations.

**Table 2:** Increased risk of extirpation (Δ*P*_extirpation_) compared to populations with no harvest, for different rates of change in carrying capacity. Population resilience is defined in terms of the unharvested per capita population growth rate referenced to maximum net productivity level (*r*_MNPL_). Harvest was calculated using a management factor (*F*_O_) of 0·75, a male‐to‐female sex ratio (*SR*) of 2 and simulated population assessments with a data precision level of 3 as defined in the text

Population resilience (*r*_MNPL_)	Percent change in carrying capacity per decade		
	0%	−7%	−14%
<−0·05	0	0	0
−0·05 to −0·025	11	10	10
−0·025 to 0	6	6	7
0 to 0·025	4	5	8
0·025 to 0·05	1	1	4
>0·05	0	0	2

To place the historical standard 4·5% harvest rate for polar bears (Taylor *et al*. 1987) in context, we continue the previous sample application of the state‐dependent management framework, assuming that values of *r* derived from unharvested survival estimates for polar bears are equivalent to *r*_MNPL_. Application of eqns 1 and 2 with these assumptions would suggest a mean harvest rate of 5·3% (95% CI = 1·7–9·7%) if applied to the lower 15th percentile of the sampling distribution for *N*. This would be equivalent to a harvest rate of approximately 5·3% × 0·85 = 4·5% if applied to the mean value of *N* (see Appendix S4). This suggests that application of the state‐dependent management framework using *F*_O_ = 0·75 and *SR* = 2, moderately conservative inputs under most conditions (Table 1), would result in a mean harvest level broadly comparable to that used for polar bears in recent decades.

## Discussion

We present a demographic model that (i) includes density‐dependent relationships based on species biology (Henle, Sarre & Wiegand 2004), (ii) produces population dynamics consistent with expectations for large mammals (Fowler 1987), and (iii) incorporates the effects of habitat loss due to climate change. Our estimate of MNPL = 0·69 was within the range of 0·50–0·85 for marine mammals (Taylor & DeMaster 1993) and similar to the range of 0·75–0·90 suggested for polar bears (Derocher & Taylor 1994). Together with the estimated ratio *r*_MNPL_/*r*_max_ = 0·82, these findings suggest that polar bears are capable of stronger compensation for human‐caused removals than would be expected under the classic logistical growth model for which MNPL = 0·5 and *r*_MNPL_/*r*_max_ = 0·5.

Our model incorporated the effects of sex, age, reproductive status and individual nutritional requirements on population dynamics. The mean ratio of the reproductive value for adult females with yearling cubs compared to 2‐year‐old females was 2·03 (95% CI = 1·83–2·28), illustrating the importance of accounting for the distribution of individuals across life‐history stages (Fig. 1). Incorporating metabolic energetic equivalent values resulted in adult males contributing approximately 30% more to density effects compared to adult females (see Appendix S2). We focused on the role of energetics in mediating population regulation because large carnivores are generally regulated by bottom‐up resource limitation (Sinclair 2003), and because Stirling & Øritsland (1995) reported covariation between the abundance of polar bears and their primary prey, ringed seals *Pusa hispida* Schreber 1775. For a particular management objective and level of risk tolerance, we expect that calculated harvest rates would be slightly lower using a model that did not include energetic effects. Future work could evaluate alternative energetic components, the potential influence of social behaviour on population regulation, and other factors that affect population dynamics including migration or genetic effects. In combination, the lower reproductive value of young bears and male bears, the higher energetic requirements of older bears and male bears, and the increased vulnerability of some sex and age classes to human‐caused mortality (Dyck 2006) have the potential to result in population sex ratios skewed towards females and equilibrium population sizes greater than what would be expected based on asymptotic population dynamics (e.g. harvest using *F*_O_ = 1 and *SR* = 2 led to populations above *MNPL* under some conditions; Table 1). A similar phenomenon of higher abundance, relative to a given resource base, has been suggested for female‐skewed populations of ungulates that result from sexual selection, resource partitioning and higher energetic requirements of males (McCullough 1999).

Our findings indicate that a 4·5% harvest rate, with a 2 : 1 male‐to‐female sex ratio in the harvest, is generally reasonable for polar bear subpopulations with medium resilience (e.g. *r*_*MNPL*_ ≈ 0·043) and data quality [e.g. CV(*N*) ≈ 0·15], provided that a state‐dependent management framework is followed and other potential sources of uncertainty are considered. Our findings also support the suggestion by Eberhardt (1990) that polar bears can sustain higher harvest rates under some conditions. This is consistent with observations that polar bear subpopulations harvested at 4·5% generally have not experienced long‐term declines when environmental conditions are productive and stable (Obbard *et al*. 2010); and with evidence that brown bears *Ursus arctos* Linnaeus 1758, which have a similar life history to polar bears, can support higher removal rates under some conditions (e.g. McLellan *et al*. 2016). Taylor, McLoughlin & Messier (2008) suggested that harvesting polar bears for maximum sustainable yield, using a 3 : 1 male‐to‐female sex ratio, could lead to a 0·25 proportion of males in the population, and depletion of adult males. We found that such reductions in males are possible at a 2 : 1 male‐to‐female sex ratio due to the effects of imperfect information and time‐lags in management. We used a proportional quasi‐extinction threshold to indirectly reflect the potential for Allee effects in the mating system at reduced densities, and suggest that future work could incorporate a mechanistic model for Allee effects (Molnár, Lewis & Derocher 2014).

Habitat loss can potentially affect populations through both density‐dependent mechanisms (e.g. increased competition for limited resources) and density‐independent mechanisms (e.g. insufficient temporal availability of resources, regardless of competition). We demonstrate the use of remote sensing data to derive a proxy metric for *K* that operates on vital rates through the density‐dependent relationships, and suggest that evaluating harvest using several trends in such a metric (here 0, 1 and 2 times the observed trend) is useful for risk assessment and scenario planning when empirical relationships between habitat and vital rates are lacking. Our model incorporated density‐independent effects by allowing populations to have variable values of *r*_max_ and by including density‐independent variation in the vital rates (e.g. due to weather fluctuations; Stirling & Lunn 1997). Also, the nonlinear density‐dependent curves resulted in nearly horizontal growth curves for densities (*N*/*K*) less than 0·5 (Fig. 2c), meaning that population responses at low densities were effectively density‐independent. This framework is sufficiently general to include a range of mechanisms by which climate change might affect populations and their response to harvest. For example, if the primary effect of habitat loss is to reduce *K*, then the harvest rate necessary to meet management objectives might not change, but the harvest level would decline with declining *N*. If the primary effect of habitat loss is to reduce *r*_max_, then the harvest rate would decline to the point that, if *r*_max_ approached 0, a harvest rate of 0 would be necessary to avoid additive mortality and accelerated population declines. We note that if, in reality, multiyear time‐lags exist between declines in *K* and reductions in vital rates, under conditions of rapidly declining *K* it is possible that human‐caused mortality would be more compensatory than in our model, effectively moving populations towards *K* while having a reduced effect on persistence.

Wildlife management decisions are often based on imprecise and infrequent population data. Although we included simulated population assessments with a single management interval of 10 years, we expect that the upper limits on *F*_O_ associated with a particular risk tolerance would increase for shorter management intervals, and vice versa. Our simulations demonstrate that the risk associated with harvest is due in large part to uncertain population data, which emphasizes the importance of periodic assessments (e.g. population studies and collection of local and traditional ecological knowledge; Vongraven *et al*. 2012). Polar bear management varies across national and regional jurisdictions (Obbard *et al*. 2010) and our approach could be extended to include other potential sources of uncertainty, such as incomplete harvest reporting. We referenced the vital rates used in the state‐dependent management framework to a population density at MNPL. For wildlife populations that are harvested near maximum sustainable yield, estimates of unharvested survival from population studies could be used to estimate a value of *r* that is close to *r*_MNPL_. The approach taken here is to infer population density relative to *K* based on knowledge of the removal rate, given that direct estimates of *K* are generally not available (Gerrodette & DeMaster 1990). For short population studies in variable environments, it may be difficult to estimate *r*_MNPL_ and therefore necessary to infer a population's capacity for growth using other demographic or ecological indicators (e.g. body condition and recruitment; Rode *et al*. 2014). The simulated population assessments could be modified to account for this additional source of uncertainty.

We evaluated harvest relative to management objectives intended to (i) maintain a population above its MNPL relative to current *K*, and (ii) have a limited negative effect on persistence. These objectives avoid the conflicting definitions of ‘sustainable harvest’ used in wildlife management (Sutherland 2001) and may reduce confusion that can result when this term is applied to populations that face current or future declines for reasons other than harvest (e.g. climate change). Objective (i) is defined relative to current conditions, which acknowledges that the effects of harvest depend on a potentially changing *K* and *r*_max_. Because populations produce maximum sustainable yield near MNPL, objective (i) also can allow wildlife managers to maximize long‐term returns while avoiding removal levels that would reduce opportunities for future use. This is important for polar bears because of the value of harvest to humans and because working cooperatively with subsistence hunters is a necessary component of on‐the‐ground management (Laidre *et al*. 2015). More broadly, this objective is consistent with the growing recognition that balancing preservation and human needs is an integral part of conservation science (Kareiva & Marvier 2012). Objective (ii) directly links management actions to targets for population viability, a common metric in conservation planning. Finally, the objectives and methods presented here require a clear statement of risk tolerance. In practice, our placeholder degree of risk tolerance would be replaced with a value based on statutory requirements, stakeholder values and the costs and benefits of use.

Our results indicate that harvest at a level designed to meet management objectives (i) and (ii) can mitigate the risk of accelerating population declines caused by habitat loss due to climate change. This requires that climate change affects populations primarily through density‐dependent mechanisms (e.g. Lunn *et al*. 2016) or that management intervals are short enough to respond to potential density‐independent reductions in *r*_MNPL_ (alternatively, that within‐interval reductions in *r*_MNPL_ are considered when deriving values of *F*_O_). The proposed state‐dependent framework implements harvest as a fraction of current population size, which is a more robust strategy than fixed‐level harvest (Quinn & Deriso 1999). A consequence of this approach is that, if populations are declining due to declining *K*, the calculated harvest level will decline as well. Our analyses suggest that the following considerations also may be important if environmental conditions are deteriorating rapidly: (i) obtaining improved population data and shortening the management interval, and (ii) reducing all human impacts, including harvest, when pre‐determined thresholds for population status are crossed (noting that, for polar bears, different types of human‐caused removals are interrelated such that defence kills may increase when subsistence harvest is decreased). Threshold harvest rules, under which there is no harvest below a lower limit on population size, can be an effective method of minimizing harvest risks while maintaining the opportunity for future use if populations rebound (Lande, Saether & Engen 1997). Although our example focuses on the subsistence harvest of polar bears, the methods presented here could be applied to other management issues (e.g. reproductive failure due to disturbance) or species and integrated with decision analysis tools to optimize trade‐offs in conservation planning (e.g. Williams & Johnson 2013).

## Authors’ contributions

E.R. and M.R. conceived the ideas and designed the methodology. E.R., R.W. and M.R. performed population projections. K.R. provided ecological theory and H.S. developed habitat metrics. E.R. led the writing of the manuscript. All authors contributed critically to interpretation and development of the drafts and gave final approval for publication.

## Data accessibility

Sea ice data and code for the matrix‐based projection model are available from Dryad Digital Repository http://dx.doi.org/10.5061/dryad.f68m0 (Regehr *et al*. 2017). Other data are included in the Supporting Information.

## Supporting information


**Fig. S1.** Contour plot of maximum net productivity level.

**Fig. S2.** Contour plot of per capita population growth rate at maximum net productivity level.

**Table S1.** Summary of published vital rates.

**Table S2.** Metabolic energetic equivalents for polar bears.

**Table S3.** Linear regressions fit to the number of ice‐covered days 1979–2013.

**Table S4.** Levels of data precision used in simulated population assessments.

**Appendix S1.** Vital rates for polar bears.

**Appendix S2.** Details on the model of density dependence.

**Appendix S3.** Methods to calculate a sea‐ice proxy for carrying capacity.

**Appendix S4.** Details on harvest and simulated population assessments.

**Appendix S5.** Summary of steps using the matrix‐based projection model.

**Appendix S6.** Software.
