# Supplementary material for: Harvesting wildlife affected by climate change: a modelling and management approach for polar bears
Source: J Appl Ecol. 2017 Mar 8;54(5):1534–43. doi: 10.1111/1365-2664.12864 (PMC5637955; doi:10.1111/1365-2664.12864)

Supporting Information for: Regehr, E.V., Wilson, R.R., Rode, K.D., Runge, M.C., & Stern, H. (2017) *Harvesting wildlife affected by climate change: a modelling and management approach for polar bears*. Journal of Applied Ecology.

Figure S1. Contour plot of maximum net productivity level (MNPL) as a function of survival and recruitment. Survival is the un-harvested adult female survival rate, averaged over the three adult female stages (4, 5, and 6) in the life cycle graph (Fig. 1). Recruitment is the number of yearlings per adult female. Vital rates are referenced to population density at MNPL. The dashed region encompasses the combinations of survival and recruitment that are feasible under the demographic model given the life cycle of polar bears (Fig. 1), our density-dependent functions of the vital rates (Fig. 2), and the assumed parameter space of 400 sets of vital rates.

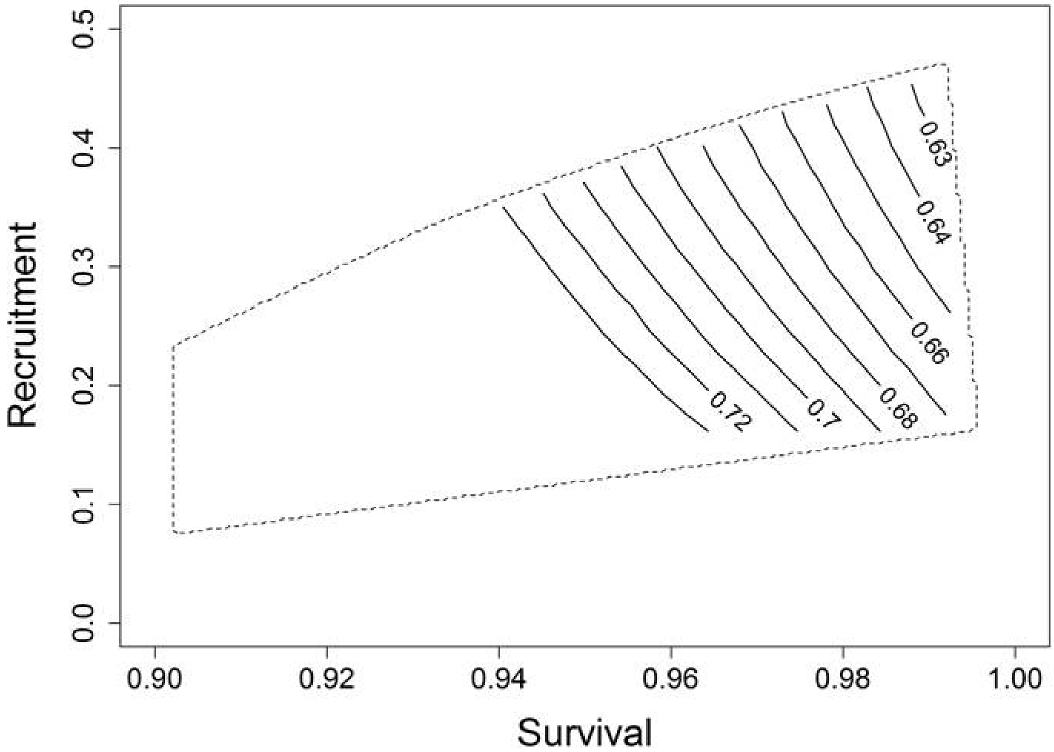

Supplement: Supplementary file 1 — Fig. S1. Contour plot of maximum net productivity level. [file JPE-54-1534-s001.pdf]
