# Supplementary material for: Harvesting wildlife affected by climate change: a modelling and management approach for polar bears
Source: J Appl Ecol. 2017 Mar 8;54(5):1534–43. doi: 10.1111/1365-2664.12864 (PMC5637955; doi:10.1111/1365-2664.12864)

Supporting Information for: Regehr, E.V., Wilson, R.R., Rode, K.D., Runge, M.C., & Stern, H. (2017) *Harvesting wildlife affected by climate change: a modelling and management approach for polar bears*. Journal of Applied Ecology.

Figure S2. Contour plot of per capita population growth rate at maximum net productivity level (r_{MNPL}) as a function of survival and recruitment. Survival is the un-harvested adult female survival rate, averaged over the three adult female stages (4, 5, and 6) in the life cycle graph (Fig. 1). Recruitment is the number of yearlings per adult female. Vital rates are referenced to population density at maximum net productivity level. The double boxplot shows the approximate location of survival and recruitment values calculated using vital rates from case studies (Table S1). The asterisk is the mean estimated per capita growth rate for case studies. Values for the Northern Beaufort Sea and Southern Hudson Bay subpopulations were not included in this mean value because estimates of un-harvested survival were not available.

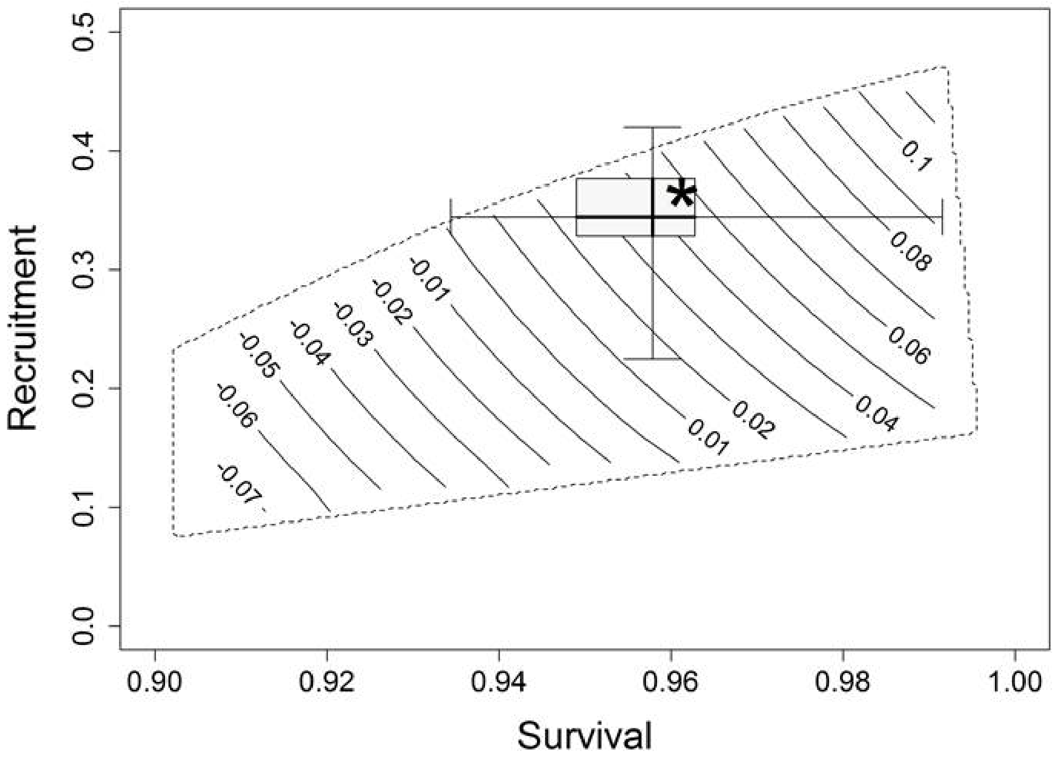

Supplement: Supplementary file 2 — Fig. S2. Contour plot of per capita population growth rate at maximum net productivity level. [file JPE-54-1534-s002.pdf]
