# Supplementary material for: Harvesting wildlife affected by climate change: a modelling and management approach for polar bears
Source: J Appl Ecol. 2017 Mar 8;54(5):1534–43. doi: 10.1111/1365-2664.12864 (PMC5637955; doi:10.1111/1365-2664.12864)
Supplement: Supplementary file 3 — Table S1. Summary of published vital rates. [file JPE-54-1534-s003.pdf]

Supporting Information for: Regehr, E.V., Wilson, R.R., Rode, K.D., Runge, M.C., & Stern, H. (2017) *Harvesting wildlife affected by climate change: a modelling and management approach for polar bears*. Journal of Applied Ecology.

Table S1. Published vital rates for polar bear subpopulations. Survival rates are estimates of un-harvested survival unless otherwise noted. Age classes are cub-of-the-year (C0), yearling (C1), subadult (2–4 yr) and adult (≥5 yr). Litter production rate is defined in Obbard *et al.* (2010)

Subpopulation	Survival								Reproduction			Reference
	Female				Male				Litter production rate		C0 litter size	
	C0	C1	Subadult	Adult	C0	C1	Subadult	Adult	5 yr	≥6 yr		
Baffin Bay	0.62	0.94	0.94	0.95	0.57	0.94	0.94	0.95	0.88	1.00	1.59	Taylor *et al.* 2005
Davis Strait-Central[1]	0.92	0.94	0.94	0.96	0.92	0.94	0.94	0.97	0.54	0.44	1.49	Peacock *et al.* 2013
Davis Strait-Northern[1]	0.89	0.92	0.92	0.95	0.89	0.92	0.90	0.94	0.54	0.44	1.49	Peacock *et al.* 2013
Davis Strait-Southern[1]	0.90	0.93	0.92	0.96	0.90	0.93	0.91	0.94	0.54	0.44	1.49	Peacock *et al.* 2013
Gulf of Boothia	0.89	0.90	0.90	0.96	0.89	0.90	0.90	0.96	0.19	0.97	1.65	Taylor *et al.* 2009
Kane Basin	0.41	0.76	0.76	1.00	0.35	0.66	0.66	1.00	0.00	0.98	1.67	Taylor *et al.* 2008a
Lancaster Sound	0.75	0.90	0.90	0.95	0.63	0.84	0.84	0.97	0.11	0.95	1.69	Taylor *et al.* 2008b

McClintock Channel	0.62	0.98	0.98	0.98	0.62	0.98	0.98	0.92	0.11	0.93	1.70	Taylor *et al.* 2006; Obbard *et al.* 2010
Northern Beaufort Sea[2]	0.52	0.33	0.91	0.91	0.52	0.32	0.83	0.83	0.28	0.88	1.76	Stirling *et al.* 2011
Norwegian Bay	0.75	0.90	0.90	0.95	0.63	0.84	0.84	0.97	0.54	0.54	1.71	Taylor *et al.* 2008b
Southern Beaufort Sea	0.34	0.93	0.93	0.97	0.34	0.90	0.90	0.96	0.44	0.44	1.72	Obbard *et al.* 2010; Regehr *et al.* 2010
Southern Hudson Bay[2]	0.73	0.72	0.92	0.91	0.59	0.58	0.86	0.86	0.97	0.97	1.58	Obbard *et al.* 2007, 2010
Viscount Melville	0.69	0.96	0.96	0.96	0.45	0.92	0.92	0.92	0.62	0.87	1.64	Taylor *et al.* 2002
Western Hudson Bay	0.73	0.73	0.92	0.93	0.71	0.71	0.94	0.94	0.26	0.79	1.54	Regehr *et al.* 2007; Obbard *et al.* 2010

[1]Peacock *et al.* (2013) estimated vital rates for three regions within the Davis Strait subpopulation.

[2]Estimates of total survival not corrected for harvest mortality.

References

Obbard, M.E., McDonald, T.L., Howe, E.J., Regehr, E.V. & Richardson, E.S. (2007) Polar Bear Population Status in Southern Hudson Bay, Canada. *U.S. Geological Survey Administrative Report*.

Obbard, M.E., Thiemann, G.W., Peacock, E. & DeBruyn, T.D. (2010) *Polar Bears: Proceedings of the 15th Working Meeting of the IUCN/SSC Polar Bear Specialist Group, Copenhagen, Denmark, 29 June - 3 July, 2009*. IUCN, Gland, Switzerland and Cambridge, UK.

Peacock, E., Taylor, M.K., Laake, J. & Stirling, I. (2013) Population ecology of polar bears in Davis Strait, Canada and Greenland. *Journal of Wildlife Management,* **77**, 463-476.

Regehr, E.V., Lunn, N.J., Amstrup, S.C. & Stirling, L. (2007) Effects of earlier sea ice breakup on survival and population size of polar bears in western Hudson Bay. *Journal of Wildlife Management,* **71**, 2673-2683.

Regehr, E.V., Hunter, C.M., Caswell, H., Amstrup, S.C. & Stirling, I. (2010) Survival and breeding of polar bears in the southern Beaufort Sea in relation to sea ice. *Journal of Animal Ecology,* **79**, 117-127.

Stirling, I., McDonald, T.L., Richardson, E.S., Regehr, E.V. & Amstrup, S.C. (2011) Polar bear population status in the northern Beaufort Sea, Canada, 1971-2006. *Ecological Applications,* **21**, 859-876.

Taylor, M.K., Laake, J., Cluff, H.D., Ramsay, M. & Messier, F. (2002) Managing the risk from hunting for the Viscount Melville Sound polar bear population. *Ursus,* **13**, 185-202.

Taylor, M.K., Laake, J., McLoughlin, P.D., Born, E.W., Cluff, H.D., Ferguson, S.H., Rosing-Asvid, A., Schweinsburg, R. & Messier, F. (2005) Demography and Viability of a Hunted Population of Polar Bears. *Arctic,* **58**, 203-214.

Taylor, M.K., Laake, J., Mcloughlin, P.D., Cluff, H.D. & Messier, F. (2006) Demographic parameters and harvest-explicit population viability analysis for polar bears in M'Clintock Channel, Nunavut, Canada. *Journal of Wildlife Management,* **70**, 1667-1673.

Taylor, M.K., Laake, J., McLoughlin, P.D., Cluff, H.D., Born, E.W., Rosing-Asvid, A. & Messier, F. (2008a) Population parameters and harvest risks for polar bears (*Ursus maritimus*) of Kane Basin, Canada and Greenland. *Polar Biology,* **31**, 491-499.

Taylor, M.K., Laake, J., McLoughlin, P.D., Cluff, H.D. & Messier, F. (2008b) Mark-recapture and stochastic population models for polar bears of the high Arctic. *Arctic,* **61**, 143-152.

Taylor, M.K., Laake, J., McLoughlin, P.D., Cluff, H.D. & Messier, F. (2009) Demography and population viability of polar bears in the Gulf of Boothia, Nunavut. *Marine Mammal Science,* **25**, 778-796.
