# Supplementary material for: Harvesting wildlife affected by climate change: a modelling and management approach for polar bears
Source: J Appl Ecol. 2017 Mar 8;54(5):1534–43. doi: 10.1111/1365-2664.12864 (PMC5637955; doi:10.1111/1365-2664.12864)
Supplement: Supplementary file 4 — Table S2. Metabolic energetic equivalents for polar bears. [file JPE-54-1534-s004.pdf]

Supporting Information for: Regehr, E.V., Wilson, R.R., Rode, K.D., Runge, M.C., & Stern, H. (2017) *Harvesting wildlife affected by climate change: a modelling and management approach for polar bears*. Journal of Applied Ecology.

Table S2. Metabolic energetic equivalents for polar bears. Age classes are cub-of-the-year (C0), yearling (C1), 2-year-old (C2), subadult (2–4 yr) and adult (≥5 yr)

C0	C1	C2		Subadult		Adult	
both sexes	both sexes	female	male	female	male	female	male
0.2	0.6	0.7	0.9	0.8	1.0	1.0	1.3
