# Supplementary material for: Harvesting wildlife affected by climate change: a modelling and management approach for polar bears
Source: J Appl Ecol. 2017 Mar 8;54(5):1534–43. doi: 10.1111/1365-2664.12864 (PMC5637955; doi:10.1111/1365-2664.12864)
Supplement: Supplementary file 5 — Table S3. Linear regressions fit to the number of ice‐covered days 1979–2013. [file JPE-54-1534-s005.pdf]

Supporting Information for: Regehr, E.V., Wilson, R.R., Rode, K.D., Runge, M.C., & Stern, H. (2017) *Harvesting wildlife affected by climate change: a modelling and management approach for polar bears*. Journal of Applied Ecology.

Table S3. Parameters for linear regressions fit to the number of ice-covered days 1979–2013. Regression parameters were used to project the number of ice-covered days forward in time using equation S2

Subpopulation	*trend* (days / year)	*sd.trend* (days / year)	*sd.annual* (days)	$ndays_{94\text{-}13}$ (days)
Chukchi Sea	-0.9	0.3	15.3	188
Southern Beaufort Sea	-2.1	0.4	24.4	252
