# Supplementary material for: Harvesting wildlife affected by climate change: a modelling and management approach for polar bears
Source: J Appl Ecol. 2017 Mar 8;54(5):1534–43. doi: 10.1111/1365-2664.12864 (PMC5637955; doi:10.1111/1365-2664.12864)
Supplement: Supplementary file 6 — Table S4. Levels of data precision used in simulated population assessments. [file JPE-54-1534-s006.pdf]

Supporting Information for: Regehr, E.V., Wilson, R.R., Rode, K.D., Runge, M.C., & Stern, H. (2017) *Harvesting wildlife affected by climate change: a modelling and management approach for polar bears*. Journal of Applied Ecology.

Table S4. Levels of data precision used in simulated population assessments. Coefficients of variation (CV) for adult female survival (σ_4) and population size (N) represent the amount of sampling uncertainty in simulated population assessments

Data precision level	Adult female survival CV(σ_4)	Population size CV(N)
true[1]	0.000	0.00
1	0.003	0.04
2	0.008	0.08
3	0.018	0.15
4	0.089	0.25

[1]Simulated population assessments used the true values of all population parameters, updated annually instead of on a 10-year management interval
