# Supplementary material for: Harvesting wildlife affected by climate change: a modelling and management approach for polar bears
Source: J Appl Ecol. 2017 Mar 8;54(5):1534–43. doi: 10.1111/1365-2664.12864 (PMC5637955; doi:10.1111/1365-2664.12864)
Supplement: Supplementary file 7 — Appendix S1. Vital rates for polar bears. [file JPE-54-1534-s007.pdf]

Supporting Information for: Regehr, E.V., Wilson, R.R., Rode, K.D., Runge, M.C., & Stern, H. (2017) *Harvesting wildlife affected by climate change: a modelling and management approach for polar bears*. Journal of Applied Ecology.

Appendix S1. Vital rates for polar bears

We obtained time-invariant estimates of vital rates for polar bears (Table S1) from the scientific literature and from proceedings of the Polar Bear Specialist Group of the International Union for the Conservation of Nature (Obbard *et al.* 2010). These vital rates were used to inform some aspects of the demographic model and to define the range of conditions under which hypothetical polar bear populations were projected forward in time, as described throughout section "Materials and methods".

We adapted the published vital rates, which were derived from capture-recapture models with different structures, to the life cycle graph (Fig. 1) using the following conventions. All survival rates represent un-harvested survival unless otherwise noted. If a study did not report a survival rate for yearling (C1) polar bears, we assumed C1 survival to be equivalent to the reported value of subadult (independent polar bear aged 2–4 years) survival. If a study reported survival for a combined C1 and subadult age class, we assumed it to be equivalent to subadult survival as defined in the life cycle graph. We used estimates of litter production rate for females >6 years (Table 3 in Obbard *et al.* 2010) as the vital rate β_4, and as the vital rate β_3 if a separate estimate of β_3 was not available. Recruitment parameters in the life cycle graph (σ_{L0}, σ_{L1}, and f) were calculated from cub-of-the year (C0) litter sex ratio, C0 litter size, C0 survival, and C1 survival as described in Appendix B of Hunter *et al.* (2007). All population projections used a constant value of 0.5 for the proportion of females in C0 litters because this is the mean estimate across subpopulations (Table 3 in Obbard *et al.* 2010) and we did not have a biological expectation for variation in this parameter. All population projections used a constant value of

0.10 for β_5 (Regehr *et al.* 2010) because this parameter is relatively unimportant to population growth (Hunter *et al.* 2007) and has not been estimated in most polar bear studies.

During the sample application of the state-dependent management framework (see SIMULATIONS), we projected populations over a wide range of biological conditions as defined by 400 sets of vital rates. This parameter space was intended to broadly represent the range of vital rates that polar bears could exhibit. It was constructed by combining 20 equal-increment intervals in natural survival, from 3% less than the minimum values to the maximum values observed in case studies (Table S1); with 20 equal-increment intervals in breeding probability and C0 litter size, from 30% less than the minimum values to the maximum values observed in case studies (Table S1). This approach assumed positive correlation among survival rates and among reproductive rates, and placed no constraints on correlation between survival rates and reproductive rates (see Appendix S3 for calculation of a correlation matrix using estimated vital rates from the Southern Beaufort Sea subpopulation).

References

Hunter, C.M., Caswell, H., Runge, M.C., Amstrup, S.C., Regehr, E.V. & Stirling, I. (2007) Polar bears in the southern Beaufort Sea II: demography and population growth in relation to sea ice conditions. *USGS Alaska Science Center, Anchorage, Administrative Report*.

Obbard, M.E., Thiemann, G.W., Peacock, E. & DeBruyn, T.D. (2010) *Polar Bears: Proceedings of the 15th Working Meeting of the IUCN/SSC Polar Bear Specialist Group, Copenhagen, Denmark, 29 June - 3 July, 2009*. IUCN, Gland, Switzerland and Cambridge, UK.

Regehr, E.V., Hunter, C.M., Caswell, H., Amstrup, S.C. & Stirling, I. (2010) Survival and breeding of polar bears in the southern Beaufort Sea in relation to sea ice. *Journal of Animal Ecology*, **79**, 117-127.
