# Supplementary material for: Harvesting wildlife affected by climate change: a modelling and management approach for polar bears
Source: J Appl Ecol. 2017 Mar 8;54(5):1534–43. doi: 10.1111/1365-2664.12864 (PMC5637955; doi:10.1111/1365-2664.12864)
Supplement: Supplementary file 8 — Appendix S2. Details on the model of density dependence. [file JPE-54-1534-s008.pdf]

Supporting Information for: Regehr, E.V., Wilson, R.R., Rode, K.D., Runge, M.C., & Stern, H. (2017) *Harvesting wildlife affected by climate change: a modelling and management approach for polar bears*. Journal of Applied Ecology.

Appendix S2. Details on the model of density dependence for polar bears

We defined relationships between vital rates and density using a form of the logistic equation: $y = (\mu - hf) + (2hf)/\{1+\exp[-m(x-c)]\}$ (eqn S1).

where y is a vital rate;

x is a dimensionless measure of density, calculated as the ratio of population size to carrying capacity (i.e. N/K);

μ is the median value of the vital rate;

hf is the half range of the vital rate, calculated as twice its standard deviation;

m is a slope coefficient; and

c is a location coefficient denoting the value of x at which the median (i.e. inflection point) occurs.

We calculated the median value (μ) for each vital rate (Fig. 1) from its maximum value and its half range (hf). The maximum of each vital rate (i.e. the value in the absence of density effects) was selected from the parameter space (see Appendix S1) for any given population projection. We used a half range (hf) of 0.05 for the vital rates σ_4, σ_6, and σ_6, which was calculated as twice the standard deviation of point estimates of adult female survival from case studies (Table S1). This assumed that total variation in survival for adult females, as estimated across polar bear subpopulations that exist under different densities and different environmental conditions, was a reasonable approximation of the amount of plasticity in this parameter. The true amount of

plasticity likely was somewhat lower due to the presence of sampling error in estimates of survival. Future analyses could evaluate this through the use of variance components.

Adult female survival is the most important determinant of population growth for many long-lived species (Eberhardt 2002). For polar bears, it is also generally estimated with higher precision than other demographic parameters. We estimated the value of *hf* for other vital rates from the value for adult females, using a linear relationship on the log-log scale between a vital rate's coefficient of variation and elasticity as calculated from the matrix model (Caswell 2001). This was a quantitative way to incorporate the hypothesis of "demographic buffering", or negative correlation between the variance of a vital rate and its importance to population growth (Pfister 1998), into the density-dependent functions. Elasticity values were not available for male survival because the life cycle graph did not reflect the role of males in reproduction. Therefore, we set *hf* for male survival equal to *hf* for female survival within the same age class.

We subjectively used a slope coefficient (m) of -7.25 in equation S1. This produced convex vital rate versus density curves for which most density-related changes occurred in the range 0.5 $<N/K<$1.5, where N is population size and K is carrying capacity. It also led to functional responses that were consistent with population dynamics theory (e.g. estimates of maximum net productivity level [MNPL] that were within the range for species with similar life history; see Results). We adjusted the location coefficient c for each vital rate, relative to the others, so that density dependence would begin to affect vital rates in the following order: subadult survival, cub-of-the-year (C0) survival, breeding probability, and finally yearling (C1) survival and adult survival (Eberhardt 2002). With the location coefficient set to c for adult survival, we set the location coefficient to 0.8c for the vital rates σ_1, σ_2, σ_3, σ_7, σ_8, and σ_9. This reflected the hypothesis that, as N/K increases, density effects will first show up in subadult

survival, because subadults are inexperienced hunters that have high energetic demands due to growth and generally are in poorer nutritional condition than other bears (Noyce & Garshelis 1994; Eberhardt 2002; Schwartz *et al.* 2006). We set the location coefficient to 0.85c for σ_{L0}, reflecting the hypothesis that C0 litter survival is the next vital rate affected. Body size and survival for C0s are related to maternal nutritional condition, which depends on food availability (Derocher & Stirling 1996) and declines in C0 survivorship have been suggested as an early response to density effects (Miller, Sellers & Keay 2003). Finally, we set the location coefficient to 0.975c for β_3 and β_4, reflecting the hypothesis that changes in breeding probability may be delayed (Noyce & Garshelis 1994) because studies of several subpopulations thought to be experiencing density effects suggest that the threshold for maternal nutritional condition, below which females fail to give birth, is low (Robbins *et al.* 2012). This potentially can lead to successful C0 litter production (i.e. relatively high β_4) followed by C0 litter loss (i.e. relatively low σ_{L0}).

The density-dependent curves generated by equation S1 were constrained to the range [0,1]. Equation S1 was applied to all vital rates in the life cycle graph (Fig. 1) except for the parameter f, the expected number of 2-year-olds arriving in stages 1 and 7. This is because f was not independent but rather was calculated as a function of C0 litter sex ratio, C0 litter size, C0 survival, and C1 survival (Appendix B in Hunter *et al.* 2010). We used a time-constant value of C0 litter size; therefore, the demographic model did not allow for potential density-dependent effects on this parameter.

We hypothesized that density dependence in polar bears is largely the result of direct (e.g. contest) and indirect (e.g. scramble) competition for nutritional resources (Derocher & Taylor 1994; Rode *et al.* 2012). However, individual polar bears vary greatly in body size and

nutritional requirements (Stirling & Øritsland 1995) and can be expected to vary in their contributions to competitive effects (e.g. a 25 kg cub will influence density-dependent regulation differently than a 500 kg adult male). To account for this variation, we calculated metabolic energetic equivalent (*mee*) values as the mean body mass for each sex and age class, raised to the 2/3 power to convert from actual body mass to metabolic body mass (White & Seymour 2003; Capellini, Vendetti & Barton 2010), and standardized to give a *mee* value of 1.0 for solitary adult females. We used data on polar bear mass from the Chukchi Sea (CS) and Southern Beaufort Sea (SB) subpopulations (Rode *et al.* 2014; U.S. Fish and Wildlife Service, unpublished data). We reduced the *mee* value for adult males by 15% to reflect the portion of male diet that consists of large prey items—for example, bearded seals *Erignathus barbatus* and beluga whales *Delphinapterus leucas*—which are less accessible to, and therefore likely do not cause competition with, polar bears in other sex and age classes (Thiemann, Iverson & Stirling 2008; Cherry *et al.* 2011). The resulting *mee* values (Table S2) represent the energetic requirements of animals relative to the requirements of solitary adult females.

We used the density-dependent functions to evaluate the demographic dynamics of polar bear populations across the 400 sets of vital rates in the parameter space (see Appendix S1), assuming a stable stage distribution and asymptotic population dynamics (Caswell 2011). Maximum net productivity level (MNPL) was negatively correlated with survival and recruitment (Fig. S1). We represent recruitment using the number of C1s per adult female, as calculated from vital rates in the life cycle graph, because this metric integrates breeding success and C0 survival, and has been used in ecological investigations for polar bears (e.g. Rode *et al.* 2014). As expected, the per capita growth rate at MNPL (r_{MNPL}) was positively correlated with survival and recruitment (Fig. S2). Observed survival and recruitment rates for polar bear

subpopulations (double boxplot in Fig. S2; based on vital rates in Table S1) provide an approximate orientation to the most relevant portions of the contour plot. Under the assumption that vital rates from case studies for polar bears were for harvested subpopulations at densities near MNPL, the corresponding median value of r_{MNPL} would be approximately 0.04 based on the location of the crosshairs in the double boxplot. For comparison, the actual mean estimate of un-harvested *r* for the case studies was 0.05 (95-percent CI = 0.02–0.09), as shown by the asterisk. The estimates reported here are conditional on several assumptions of the demographic model, particularly the form of our density-dependent functions. We used these estimates of un-harvested *r* to guide the levels of population resilience that were evaluated during simulations to identify threshold values for parameters in the state-dependent management framework (see SIMULATIONS). Specifically, these simulations were performed using sets of vital rates from the parameter space that resulted in asymptotic r_{MNPL} = 0.015, 0.043, and 0.085; defined as populations with low, medium, and high resilience, respectively.

References

Capellini, I., Venditti, C. & Barton, R.A. (2010) Phylogeny and metabolic scaling in mammals. *Ecology,* **91**, 2783-2793.

Cherry, S.G., Derocher, A.E., Hobson, K.A., Stirling, I. & Thiemann, G.W. (2011) Quantifying dietary pathways of proteins and lipids to tissues of a marine predator. *Journal of Applied Ecology,* **48**, 373-381.

Caswell, H. (2001) *Matrix Population Models*, 2nd edn. Sinauer Associates Inc., Sunderland, Massachusetts.

Derocher, A.E. & Taylor, M.K. (1994) Density-dependent population regulation of polar bears. *Density-dependent population regulation in black, brown, and polar bears* (ed M.K. Taylor), pp. 25-30. International Conference on Bear Research and Management Monograph Series No. 3.

Derocher, A.E. & Stirling, I. (1996) Aspects of survival in juvenile polar bears. *Canadian Journal of Zoology,* **74**, 1246-1252.

Eberhardt, L.L. (2002) A paradigm for population analysis of long-lived vertebrates. *Ecology,* **83**, 2841-2854.

Hunter, C.M., Caswell, H., Runge, M.C., Regehr, E.V., Amstrup, S.C. & Stirling, I. (2010) Climate change threatens polar bear populations: a stochastic demographic analysis. *Ecology,* **91**, 2883-2897.

Miller, S.D., Sellers, R.A. & Keay, J.A. (2003) Effects of hunting on brown bear cub survival and litter size in Alaska. *Ursus,* **14**, 130-152.

Noyce, K.V. & Garshelis, D.L. (1994) Body size and blood characteristics as indicators of condition and reproductive performance in black bears. *International Conference of Bear Research and Management,* **91**, 481-496.

Pfister, C.A. (1998) Patterns of Variance in Stage-Structured Populations: Evolutionary Predictions and Ecological Implications. *Proceedings of the National Academy of Sciences of the United States of America,* **95**, 213-218.

Robbins, C.T., Ben-David, M., Fortin, J.K. & Nelson, O.L. (2012) Maternal condition determines birth date and growth of newborn bear cubs. *Journal of Mammalogy,* **93**, 540-546.

Rode, K.D., Peacock, E., Taylor, M., Stirling, I., Born, E.W., Laidre, K.L. & Wiig, Ø. (2012) A tale of two polar bear populations: ice habitat, harvest, and body condition. *Population Ecology,* **54**, 3-18.

Rode, K.D., Regehr, E.V., Douglas, D.C., Durner, G., Derocher, A.E., Thiemann, G.W. & Budge, S.M. (2014) Variation in the response of an Arctic top predator experiencing habitat loss: feeding and reproductive ecology of two polar bear populations. *Global Change Biology,* **20**, 76-88.

Schwartz, C.C., Haroldson, M.A., White, G.C., Harris, R.B., Cherry, S., Keating, K.A., Moody, D. & Servheen, C. (2006) Temporal, Spatial, and Environmental Influences on the Demographics of Grizzly Bears in the Greater Yellowstone Ecosystem. *Wildlife Monographs*, **161**, 1-68.

Stirling, I. & Øritsland, N.A. (1995) Relationships between estimates of ringed seal (*Phoca hispida*) and polar bear (*Ursus maritimus*) populations in the Canadian Arctic. *Canadian Journal of Fisheries and Aquatic Sciences*, **52**, 2594-2612.

Thiemann, G.W., Iverson, S.J. & Stirling, I. (2008) Polar bear diets and Arctic marine food webs: insights from fatty acid analysis. *Ecological Monographs,* **78,** 591-613.

White, C.R. & Seymour, R.S. (2003) Mammalian basal metabolic rate is proportional to body mass(2/3). *Proceedings of the National Academy of Sciences of the United States of America,* **100**, 4046-4049.
