# Supplementary material for: Harvesting wildlife affected by climate change: a modelling and management approach for polar bears
Source: J Appl Ecol. 2017 Mar 8;54(5):1534–43. doi: 10.1111/1365-2664.12864 (PMC5637955; doi:10.1111/1365-2664.12864)
Supplement: Supplementary file 9 — Appendix S3. Methods to calculate a sea‐ice proxy for carrying capacity. [file JPE-54-1534-s009.pdf]

Supporting Information for: Regehr, E.V., Wilson, R.R., Rode, K.D., Runge, M.C., & Stern, H. (2017) *Harvesting wildlife affected by climate change: a modelling and management approach for polar bears*. Journal of Applied Ecology.

## Appendix S3. Methods to calculate a sea-ice proxy for carrying capacity and to represent the effects of environmental variation on vital rates

The Arctic marine environment is highly variable (Walsh 2008), which can influence persistence even when population sizes are large (Boyce, Haridas & Lee 2006). Given that the primary threat to polar bears is habitat loss due to climate change (Atwood *et al.* 2016), methods to evaluate harvest and persistence should consider relationships between environmental variation and vital rates. We represented environmental variation as the additive effects of two components: (1) temporal variation in carrying capacity ($K$), which operated on vital rates through the density-dependent functions; and (2) density-independent temporal variation in vital rates.

We obtained passive microwave satellite data of daily sea-ice concentration for 1979–2013 (Cavalieri *et al.* 1996, updated yearly) within the Chukchi Sea (CS) and Southern Beaufort Sea (SB) polar bear subpopulation boundaries (Obbard *et al.* 2010). The sea-ice concentrations were generated using the National Aeronautics and Space Administration Team algorithm and were provided in a polar stereographic projection with a nominal grid cell size of 25 × 25 km. Within each subpopulation boundary, we first determined the mean area covered by sea ice during March of each year (i.e. the sea ice maximum), for 1979–2013. Area covered by sea ice was calculated as the sum of grid cell area multiplied by grid cell concentration, over all grid cells with ≥15% sea ice concentration. Next, we determined the number of days each year when the area of ice cover was greater than 50% of the March area, hereafter referred to as "ice-covered days." We then used linear regression to estimate the trend in the number of ice-covered days (*trend*) and the standard deviation of the trend (*sd.trend*). The standard deviation of the

residuals (*sd.annual*) provided a measure of interannual variability. Finally, we calculated the mean number of ice-covered days for the period 1994–2013 ($ndays_{94\text{-}13}$) to serve as a baseline for projections, because this period was used to represent current ecological conditions for polar bears in a comparative analysis of body condition and recruitment between the CS and SB subpopulations (Rode et al. 2014). Based on these results (Table S3), we projected the number of ice-covered days forward in time using a gamma distribution to ensure that values were greater than zero:

$$ndays_t \sim \text{Gamma}\left(shape = \frac{\left(ndays_{94-13} + \widehat{trend} \times t\right)^2}{sd.annual^2},\ \ rate = \frac{\left(ndays_{94-13} + \widehat{trend} \times t\right)}{sd.annual^2}\right) \text{(eqn S2)}$$

where $ndays_t$ is the number of ice-covered days in year $t$;

$t$ is the number of years from the beginning of the projection; and

$\widehat{trend}$ is a random draw, at the beginning of each projection, from a normal distribution with mean *trend* and standard deviation *sd.trend*.

We then calculated the proportional change in *ndays* over time as $\kappa = ndays_t / ndays_{94\text{-}13}$. Thus, the dimensionless metric $\kappa$ reflects proportional changes in the duration of the ice-covered period. Carrying capacity at year $t$ was calculated as $K(t) = K(t{=}1) \times \kappa(t)$, and operated on vital rates through the density-dependent curves. The resulting values of $K(t)$ represent a proxy for true environmental carrying capacity, which is unknown, and were used to explore the demographic consequences of variation in the Arctic environment. A detailed presentation of sea-ice metrics used in polar bear studies is provided by Stern & Laidre (2016).

Density-independent variation in vital rates (Fig. 1) for polar bears can arise from weather fluctuations (Stirling & Smith 2004), changes in prey populations (Stirling & Lunn 1997), or other factors. We subjectively assumed that such variation constitutes 25% of total uncertainty (i.e. temporal variation plus sampling uncertainty) in estimated vital rates (Table S1), following the example of Taylor *et al.* (2002). The resulting estimate of the temporal coefficient of variation for $\sigma_4$, the most important vital rate, was 0.014. Because population persistence is influenced by both the magnitude and correlation structure of variation (Doak *et al.* 2005), we derived a correlation matrix from annual estimates of vital rates for the SB subpopulation (Regehr *et al.* 2010), and used this correlation matrix for all population projections. This analysis suggested that there was nearly complete correlation among survival rates (e.g. a mean correlation coefficient of 0.99 between $\sigma_4$ and other survival rates) and positive correlation between survival and reproductive rates (e.g. 0.67 between $\sigma_4$ and $\beta_4$).

We used correlated non-normal distributions to capture density-independent temporal variation in vital rates. To simulate these distributions, we used methods described in Morris & Doak (2002, p. 282–287) to transform correlated standard normal deviates into correlated values, from either a multivariate beta distribution (for all vital rates in Fig.1 except the recruitment parameter *f*) or a stretched beta distribution (for cub-of-the-year [C0] litter size, which was used to calculate *f*). For the vital rates $\beta_3$ and $\beta_4$, shape parameters for the beta distribution were occasionally inestimable due to high variances. In such cases, these vital rates were randomly selected from a uniform distribution with a range [0, 0.5] or [0.5, 1.0] depending on whether the standard normal deviate was less than or greater than 0.5, respectively.

**References**

Atwood, T.C., Marcot, B.G., Douglas, D.C., Amstrup, S.C., Rode, K.D., Durner, G.M. & Bromaghin, J.F. (2016) Forecasting the relative influence of environmental and anthropogenic stressors on polar bears. *Ecosphere* **7**, e01370.10.1002/ecs2.1370.

Boyce, M.S., Haridas, C.V. & Lee, C.T. (2006) Demography in an increasingly variable world. *Trends in Ecology & Evolution*, **21**, 141-148.

Cavalieri, D. J., C. L. Parkinson, P. Gloersen, & H. J. Zwally. 1996, updated yearly. Sea Ice Concentrations from Nimbus-7 SMMR and DMSP SSM/I-SSMIS Passive Microwave Data, Version 1. Boulder, Colorado USA. NASA National Snow and Ice Data Center Distributed Active Archive Center, doi: http://dx.doi.org/10.5067/8GQ8LZQVL0VL.

Doak, D.F., Morris, W.F., Pfister, C., Kendall, B.E. & Bruna, E.M. (2005) Correctly Estimating How Environmental Stochasticity Influences Fitness and Population Growth. *American Naturalist*, **166**, E14-E21.

Morris, W.F. & Doak, D.F. (2002) *Quantitative Conservation Biology: Theory and Practice of Population Viability Analysis*. Sinauer Associates, Inc., Sunderland, Massachusetts.

Obbard, M.E., Thiemann, G.W., Peacock, E. & DeBruyn, T.D. (2010) *Polar Bears: Proceedings of the 15th Working Meeting of the IUCN/SSC Polar Bear Specialist Group, Copenhagen, Denmark, 29 June - 3 July, 2009*. IUCN, Gland, Switzerland and Cambridge, UK.

Regehr, E.V., Hunter, C.M., Caswell, H., Amstrup, S.C. & Stirling, I. (2010) Survival and breeding of polar bears in the southern Beaufort Sea in relation to sea ice. *Journal of Animal Ecology*, **79**, 117-127.

Rode, K.D., Regehr, E.V., Douglas, D.C., Durner, G., Derocher, A.E., Thiemann, G.W. & Budge, S.M. (2014) Variation in the response of an Arctic top predator experiencing habitat loss: feeding and reproductive ecology of two polar bear populations. *Global Change Biology,* **20**, 76-88.

Stern, H.L. & Laidre, K.L. (2016) Sea-ice indicators of polar bear habitat. *The Cryosphere* **10**, 2027-2041.

Stirling, I. & Lunn, N.J. (1997) Environmental fluctuations in Arctic marine ecosystems as reflected by variability in reproduction of polar bears and ringed seals. *Ecology of Arctic Environments* (eds S.J. Woodin & M. Marquiss), pp. 167-181. Blackwell Scientific Publications, Oxford.

Stirling, I. & Smith, T.G. (2004) Implications of warm temperatures and an unusual rain event for the survival of ringed seals on the coast of southeastern Baffin Island. *Arctic,* **57**, 59-67.

Taylor, M.K., Laake, J., Cluff, H.D., Ramsay, M. & Messier, F. (2002) Managing the risk from hunting for the Viscount Melville Sound polar bear population. *Ursus,* **13**, 185-202.

Walsh, J.E. (2008) Climate of the Arctic Marine Environment. *Ecological Applications,* **18**, S3-S22.
