# Supplementary material for: Harvesting wildlife affected by climate change: a modelling and management approach for polar bears
Source: J Appl Ecol. 2017 Mar 8;54(5):1534–43. doi: 10.1111/1365-2664.12864 (PMC5637955; doi:10.1111/1365-2664.12864)
Supplement: Supplementary file 10 — Appendix S4. Details on harvest and simulated population assessments. [file JPE-54-1534-s010.pdf]

Supporting Information for: Regehr, E.V., Wilson, R.R., Rode, K.D., Runge, M.C., & Stern, H. (2017) *Harvesting wildlife affected by climate change: a modelling and management approach for polar bears*. Journal of Applied Ecology.

Appendix S4. Details on harvest and simulated population assessments

The reproductive value of polar bears varies with sex and age (Hunter *et al.* 2007). Furthermore, subsistence harvests generally are sex-selective, and young polar bears are more likely than older bears to be killed by humans due to behavioural and nutritional factors (Derocher, Stirling & Calvert 1997; Dyck 2006). To reflect this variation, the demographic and management model included stage-specific harvest vulnerabilities (e.g. Taylor *et al.* 2005) so that removals could be allocated accurately over life cycle stages. We estimated harvest vulnerability for subadults compared to adults using the stage distribution for the Southern Beaufort Sea (SB) subpopulation from 2001 to 2006 (Regehr *et al.* 2010) and hunter-reported age classes of harvested bears during the same period (U.S. Fish and Wildlife Service, unpublished data). The resulting harvest vulnerabilities were similar to those estimated using 28 years of data for the western Hudson Bay subpopulation (Derocher, Stirling & Calvert 1997). We set harvest vulnerability to 0.1 (relative to single adult females in stage 4) for stages 5 and 6, because taking adult females with dependent young is restricted and occurs rarely in most subpopulations (e.g. Brower *et al.* 2002).

Simulated population assessments were used to incorporate the effects on management decisions of imprecise population data and time lags in management. Simulated population assessments were performed on a 10-year management interval, which is broadly representative for relatively well-studied polar bear subpopulations, noting that management intervals vary over time and across national and regional jurisdictions (Obbard *et al.* 2010). At the beginning of each management interval, we simulated a population assessment to generate the input parameters for equations 1 and 2 used to calculate harvest level (see STATE-DEPENDENT MANAGEMENT FRAMEWORK). Per capita population growth rate at maximum net productivity level (r_{MNPL})

was estimated from correlated random vital rates, selected from a multivariate beta distribution using methods described in Appendix S3. The mean values in the multivariate beta distribution were the true vital rates at a population density equal to maximum net productivity level (MNPL), as determined from the density-dependent functions for the vital rates. A random value for estimated population size (N) was selected from a normal distribution. The mean value in the normal distribution was the average true value of N for the projected population over the preceding five annual increments. We used the average true value of N at $t = 1$ to specify harvest during the first management interval. Random draws were performed using a correlation coefficient of 1 across the vital rates and N. The amount of sampling error in the simulated population assessment (i.e. the variances in the beta and normal distributions) was based on 75% of total uncertainty in parameter estimates from case studies (Table S1). This was complementary to the previous assumption that process variation constituted 25% of total uncertainty in estimates of vital rates. Uncertainty in estimates of N was obtained from the most recent case studies for each polar bear subpopulation as summarized in the Status Table published by the Polar Bear Specialist Group of the International Union for Conservation of Nature (*http://pbsg.npolar.no/en/status/status-table.html*, accessed March 30, 2016). We considered four levels of data precision in the simulated population assessments (Table S4) based on the 1st, 10th, 50th, and 99th percentiles of estimated sampling uncertainty. Thus, our simulated population assessments broadly reflected the observed range of data precision in case studies for polar bears.

We placed the historical standard 4.5% harvest rate for polar bears (Taylor *et al.* 1987) in the context of the state-dependent management framework, by continuing the sample application using $F_O = 0.75$ and $SR = 2$, and assuming that values of r derived from un-harvested survival

estimates from case studies (Table S1) were equivalent to r_{MNPL}. This assumption is likely broadly valid given that many polar bear subpopulations have been harvested for maximum sustainable yield. However, the historic standard 4.5% harvest rate has generally been applied to the best estimate (i.e. mean value) of *N*, whereas our sample application of the state-dependent management framework uses a lower percentile of the sampling distribution for *N*. We adjust for this difference by assuming a normal distribution for estimates of *N*, with a coefficient of variation of 0.15, corresponding to data precision level 3. The lower 15th percentile of such a distribution is approximately 0.85 of the mean value. Thus, the mean harvest rate from the state-dependent management framework would be approximately 5.3% × 0.85 = 4.5% if applied to the mean value of *N*.

References

Brower, C.D., Carpenter, A., Branigan, M.L., Calvert, W., Evans, T., Fischbach, A.S., Nagy, J.A., Schliebe, S. & Stirling, I. (2002) The Polar Bear Management Agreement for the Southern Beaufort Sea: an Evaluation of the First Ten Years of a Unique Conservation Agreement. *Arctic*, **55**, 362-372.

Derocher, A.E., Stirling, I. & Calvert, W. (1997) Male-biased harvesting of polar bears in western Hudson Bay. *Journal of Wildlife Management*, **61**, 1075-1082.

Dyck, M.G. (2006) Characteristics of Polar Bears Killed in Defense of Life and Property in Nunavut, Canada, 1970-2000. *Ursus*, **17**, 52-62.

Hunter, C.M., Caswell, H., Runge, M.C., Amstrup, S.C., Regehr, E.V. & Stirling, I. (2007) Polar bears in the southern Beaufort Sea II: demography and population growth in relation to sea ice conditions. *USGS Alaska Science Center, Anchorage, Administrative Report*.

Obbard, M.E., Thiemann, G.W., Peacock, E. & DeBruyn, T.D. (2010) *Polar Bears: Proceedings of the 15th Working Meeting of the IUCN/SSC Polar Bear Specialist Group, Copenhagen, Denmark, 29 June - 3 July, 2009*. IUCN, Gland, Switzerland and Cambridge, UK.

Regehr, E.V., Hunter, C.M., Caswell, H., Amstrup, S.C. & Stirling, I. (2010) Survival and breeding of polar bears in the southern Beaufort Sea in relation to sea ice. *Journal of Animal Ecology*, **79**, 117-127.

Taylor, M.K., DeMaster, D.P., Bunnell, F.L. & Schweinsburg, R.E. (1987) Modeling the sustainable harvest of female polar bears. *Journal of Wildlife Management*, **51**, 811-820.

Taylor, M.K., Laake, J., McLoughlin, P.D., Born, E.W., Cluff, H.D., Ferguson, S.H., Rosing-Asvid, A., Schweinsburg, R. & Messier, F. (2005) Demography and Viability of a Hunted Population of Polar Bears. *Arctic*, **58**, 203-214.
