# Supplementary material for: Harvesting wildlife affected by climate change: a modelling and management approach for polar bears
Source: J Appl Ecol. 2017 Mar 8;54(5):1534–43. doi: 10.1111/1365-2664.12864 (PMC5637955; doi:10.1111/1365-2664.12864)
Supplement: Supplementary file 11 — Appendix S5. Summary of steps using the matrix‐based projection model. [file JPE-54-1534-s011.pdf]

Supporting Information for: Regehr, E.V., Wilson, R.R., Rode, K.D., Runge, M.C., & Stern, H. (2017) *Harvesting wildlife affected by climate change: a modelling and management approach for polar bears*. Journal of Applied Ecology.

Appendix S5. Summary of steps using the matrix-based projection model

We projected hypothetical polar bear populations forward in time to evaluate the effects of different biological and management conditions, and to calculate inputs to the state-dependent management framework. At each time step $t = 2,3,\ldots k$ the following operations were performed. First, populations were projected forward 1 year using a stage-structured matrix model: $\boldsymbol{n}(t+1) = \boldsymbol{A}(t) \times \boldsymbol{n}(t)$, where $\boldsymbol{n}(t)$ is a stage distribution vector representing the number of animals in each life cycle stage at time step t, and $\boldsymbol{A}(t)$ is a 10×10 projection matrix (Caswell 2001). Entries in $\boldsymbol{A}(t)$ were defined in terms of vital rates in the life cycle graph (Fig. 1). Second, harvest was allocated among stages using a multinomial distribution with the probability for each stage calculated as the product of its proportional stage distribution and harvest vulnerability. Third, population density (represented by population size [N] divided by carrying capacity [K]) was calculated by summing metabolic energetic equivalent (*mee*) values across animals in the population, then dividing by the total *mee* values available at carrying capacity. The survival and reproductive rates corresponding to this density were determined from the density-dependent curves. Fourth, these vital rates were subject to density-independent variation, as described in Appendix S3. Finally, the resulting vital rates were used to construct a projection matrix for the next time step $\boldsymbol{A}(t+1)$.

For a given population projection, inputs included maximum values of vital rates (i.e. survival and reproductive rates at low density), the starting value of K expressed as a number of animals, and the rate of human-caused removals. The equilibrium population size at maximum net productivity level (MNPL) at any given time step was calculated as $\text{MNPL}(t) \approx 0.69 \times K(t)$; where K is carrying capacity expressed as a number of animals, and referenced to time (t)

because projections included temporal variation in *K*; and 0.69 is the mean percentage of *K* at which population growth results in the greatest net increment in abundance (see Results) based on our density-dependent curves. Populations started at *t*=1 with a stable stage distribution at a population size equal to MNPL. Density-dependent curves for the vital rates are described in Appendix S2.

During allocation of harvest among stages, if the target harvest level exceeded the number of bears in a particular stage (Fig. 1), excess harvest was allocated to the more populous stages 4 or 10, for females or males, respectively. If the male segment of the population was exhausted, which happened occasionally for male-biased removals at high rates, excess male removals were allocated to stage 4 females.

During population projections we defined persistence as maintaining a population size greater than a quasi-extinction threshold of 15% of starting population size at each time step in the projection. We used a proportional quasi-extinction threshold because of potential Allee effects caused by reduced breeding encounters and mating success at low population densities (Molnár *et al.* 2008). Low population densities could result if *N* declines while the amount of habitat during the breeding season remains stable. This could occur for polar bears because they mate in April and May (Amstrup 2003), and projected sea-ice loss at that time of the year is relatively small (Stroeve *et al.* 2012).

During simulations, inputs to the state-dependent management framework were calculated using a placeholder degree of risk tolerance for the purpose of illustration; this degree of risk tolerance is not suggested as a management objective for polar bears. Specifically, we inferred an upper limit on the probability of a population remaining above MNPL (i.e. $P_{<MNPL}$) that corresponds to a harvest level that, absent all other threats, would decrease the probability of

persistence by 10%. We estimated this limit by plotting (1 – the probability of persistence [i.e., $P_{persist}$]) as a function of $P_{<MNPL}$, selecting a polynomial fit using Akaike's Information Criterion and standard regression methods (Zar 2010), and identifying the value of $P_{<MNPL}$ for which the upper 95% prediction interval corresponded to $(1 - P_{persist}) = 0.10$.

References

Amstrup, S.C. (2003) Polar Bear (*Ursus maritimus*). *Mammals of North America: Biology, Management, and Conservation* (eds G.A. Feldhamer, B.C. Thompson & J.A. Chapman), pp. 587-610. John Hopkins University Press, Baltimore, Maryland.

Caswell, H. (2001) *Matrix Population Models*, 2nd edn. Sinauer Associates Inc., Sunderland, Massachusetts.

Molnár, P.K., Derocher, A.E., Lewis, M.A. & Taylor, M.K. (2008) Modelling the Mating System of Polar Bears: a Mechanistic Approach to the Allee Effect. *Proceedings of the Royal Society B-Biological Sciences*, **275**, 217-226.

Stroeve, J.C., Kattsov, V., Barrett, A., Serreze, M., Pavlova, T., Holland, M. & Meier, W.N. (2012) Trends in Arctic sea ice extent from CMIP5, CMIP3 and observations. *Geophysical Research Letters* **39**. L16502.

Zar, J.H. (2010) *Biostatistical analysis*, 5th edn. Pearson Prentice Hall, Upper Saddle River, New Jersey.
