# Supplementary material for: Harvesting wildlife affected by climate change: a modelling and management approach for polar bears
Source: J Appl Ecol. 2017 Mar 8;54(5):1534–43. doi: 10.1111/1365-2664.12864 (PMC5637955; doi:10.1111/1365-2664.12864)
Supplement: Supplementary file 12 — Appendix S6. Software. [file JPE-54-1534-s012.pdf]

Supporting Information for: Regehr, E.V., Wilson, R.R., Rode, K.D., Runge, M.C., & Stern, H. (2017) *Harvesting wildlife affected by climate change: a modelling and management approach for polar bears*. Journal of Applied Ecology.

Appendix S6. Software and cloud computing

The demographic and management model was built using the R computing language (version R 3.1.0; The R Project for Statistical Computing; *http://www.r-project.org*). The package "popbio" was used to analyze matrix models (Stubben & Milligan 2007). Computations were performed on the Amazon Elastic Compute Cloud (*http://aws.amazon.com/ec2/*) where we ran R using an Amazon Machine Image for RStudio Server (RStudio 2014) developed by L. Aslett (*http://www.louisaslett.com/RStudio_AMI/*).

References

RStudio (2014) RStudio: Integrated development environment for R. Boston, MA.

Stubben, C. & Milligan, B. (2007) Estimating and Analyzing Demographic Models Using the popbio Package in R. *Journal of Statistical Software*, **22**, 1-23.
